# Suppression of *GhGLU19* encoding β-1,3-glucanase promotes seed germination in cotton

**DOI:** 10.1186/s12870-022-03748-w

**Published:** 2022-07-22

**Authors:** Haitang Wang, Xuesong Zhou, Chuchu Liu, Weixi Li, Wangzhen Guo

**Affiliations:** State Key Laboratory of Crop Genetics and Germplasm Enhancement, Cotton Germplasm Enhancement and Application Engineering Research Center (Ministry of Education), Nanjing Agricultural University, Nanjing, 210095 China

**Keywords:** β-1,3-glucanase, Callose, Inner integument, ABA biosynthesis, Seed germination, *Gossypium hirsutum*

## Abstract

**Background:**

In eudicots, germination begins with water uptake by the quiescent dry seed and is greatly related to the permeability of micropyle enriched callose layers. Once imbibition starts, seeds undergo a cascade of physiological, biochemical, and molecular events to initiate cellular activities. However, the effects of callose on water uptake and following seed metabolic events during germination are largely unknown. Cotton (*Gossypium hirsutum*) is a eudicot plant with natural fiber and edible oil production for humans. Here, we addressed this question by examining the role of *GhGLU19*, a gene encoding β-1,3-glucanase, in cotton seed germination.

**Results:**

GhGLU19 belongs to subfamily B and was expressed predominately in imbibed seeds and early seedlings. Compared to wild type, *GhGLU19*-suppressing and *GhGLU19*-overexpressing transgenic cotton lines showed the higher and lower seed germination percentage, respectively. Callose was enriched more at inner integument (ii) than that in embryo and seed coat in cotton seeds. In *GhGLU19*-suppressing lines, callose at ii of cotton seeds was greatly increased and brought about a prolonged water uptake process during imbibition. Both proteomic and transcriptomic analysis revealed that contrary to *GhGLU19*-overexpressing lines, the glycolysis and pyruvate metabolism was decreased, and abscisic acid (ABA) biosynthesis related genes were downregulated in imbibed seeds of *GhGLU19*-suppressing lines. Also, endogenous ABA was significantly decreased in *GhGLU19*-suppressing line while increased in *GhGLU19*-overexpressing line.

**Conclusions:**

Our results demonstrate that suppression of *GhGLU19* improves cotton seed germination via accumulating callose of inner integument, modulating glycolysis and pyruvate metabolism, and decreasing ABA biosynthesis. This study provides a potential way for improving germination percentage in cotton seed production, and other eudicot crops.

**Supplementary Information:**

The online version contains supplementary material available at 10.1186/s12870-022-03748-w.

## Background

Beta-1,3-glucanases (E.C. 3.2.1.39), a member of glycoside hydrolase family 17, catalyze the hydrolysis of β-1,3-glucans. The genes encoding β-1,3-glucanases are a big family which widely exist in many organisms, including bacteria, fungi, viruses, animals and plant kingdoms [1]. Genome-wide identification of β-1,3-glucanase genes have been performed in several plant species, including tobacco [2, 3], soybean [4], tomato [5], *Arabidopsis* [6], maize [7], rice [8], and cotton [9]. With great functional diversity, β-1,3-glucanases are well known as pathogenesis-related proteins, they also play important roles in plant physiological and developmental processes, including microsporogenesis, pollen tube development, and regulation of plasmodesmata signaling and abscission [6, 8, 10].

Seeds play an essential role during plant development and growth cycle, which consist of protective seed coat layers of dead cells, delicate embryo and the nutritive tissues. In many eudicot plants, seed coats contain two integuments, termed as testa and inner integument (ii). Callose layer was observed as a perisperm-endosperm enveloped in ii, and enriched at the micropylar region of seeds [11]. In *Geranium carolinianum*, multiple layers of rectangular-shaped parenchyma cells contribute to the formation of a dome-shaped structure at the micropyle, which is necessary to maintain water impermeability [12]. In general, seed germination proceeds following process: initial phase of water uptake by the dry seeds, testa rupture and subsequent endosperm rupture and radicle growth [13]. The increase in the total water content of seeds follows a classic triphasic model, which comprises three phases (I, II and III) of water uptake. The initial and final rapid water uptake periods (I and III, respectively) are intervened by the slow water uptake phase II [14]. Physically, certain water uptake time is necessary because imbibition metabolism must be initiated to permit the recovery from structural damage caused by maturation drying and oxidation [15, 16]. The expression of glucanase encoding genes were observed during germination of pea [17], tomato [5], cucumber [18], and tobacco [1]. A chimeric Class I isoforms of β-1,3-glucanase (βGLU I) gene regulated by an ABA-inducible *Cat1* promoter was transformed into tobacco, and found that endosperm rupture of mature seeds during imbibition were promoted in *βGLU I*-overexpressing lines [10]. These cases indicated that β-1,3-glucanase played a key role during seed germination.

Cotton (*Gossypium hirsutum*) is the most important textile and oil crop worldwide. With the rapid application of mechanized cultivation and harvest, the rapid and uniform seed germination will be particularly important to maximize crop yield potential [19]. In addition to fibers, cottonseed consists of embryo and two integuments of outer and inner seed coat, where embryo composed with differentiated meristems, radicle, and cotyledons. During germination, radicle penetrated from micropyle, which requires weakening the endosperm cap tissue [5]. The micropylar endosperm in cotton seed had approximately 10 layers’ cells formed a cap-like structure covering radicle tip [20]. Genome-wide identification revealed that cotton β-1,3-glucanase genes can be classified into eight subfamilies (A-H) with functional diversity [9]. However, it remains unclear how β-1,3-glucanase and the endosperm related metabolic processes function during seed germination. In this study, we verified the function of a cotton β-1,3-glucanase encoding gene, *GhGLU19*, during seed germination. *GhGLU19* belonged to subfamily B and was preferentially expressed in imbibed seeds and early seedlings. Using *GhGLU19* overexpression and antisense transgenic cotton lines, we performed the phenotype investigation, biophysical and biochemical analysis, isobaric tags for relative and absolute quantitation (iTRAQ) assay, and transcriptome comparison to elucidate the functional role of *GhGLU19* in seed germination. The results demonstrated that suppression of *GhGLU19* promotes seed germination. Compared to wild type and *GhGLU19*-overexpressing cotton, callose in ii was greatly increased, glycolysis and pyruvate metabolism and endogenous ABA level was significantly decreased in *GhGLU19*-suppressing seeds. The study provides a potential way for improving the seed germination percentage in cotton seed production, and other eudicot crops.

## Results

### *GhGLU19* encoded β-1,3-glucanase and was preferentially expressed during seed germination

**Fig. 1:**
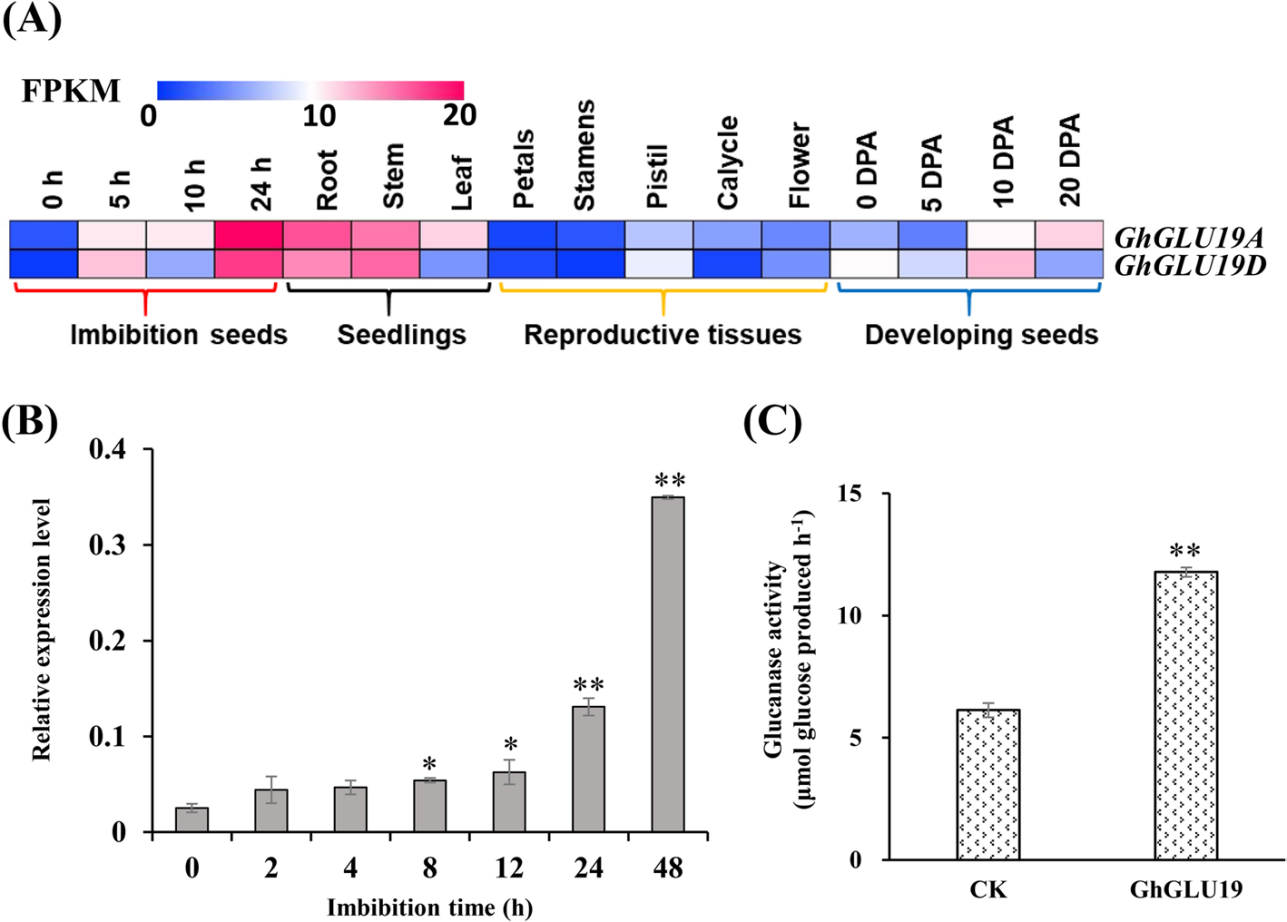
The expression profile and enzyme activity of GhGLU19. **A** The expression profiling of *GhGLU19A* (*Gh_A04G0109*) and *GhGLU19D* (*Gh_D05G3612*) in tissues of *G. hirsutum* acc. TM-1. Imbibed seeds at 0, 5, 10, 24 h, root, stem, leaf, petal, stamen, pistil, calycle, flower, and ovules at 0, 5, 10 and 20 days post anthesis (DPA) were used for transcriptional analysis. The FPKM were used to calculate the expression level of *GhGLU19A* and *GhGLU19D*, respectively. Colored squares indicated expression levels from 0 (blue) to 20 (red). **B** The expression level of *GhGLU19* in imbibition seeds at 2, 4, 8, 12, 24 and 48 h after imbibition compared with that at 0 h by qRT-PCR analysis. Three independent biological replicates were analyzed with three technical repeats. Student’s *t*-test, * *P* < 0.05, ** *P* < 0.01. **C** Enzyme activity of *GhGLU19* was detected by the spectrophotometric method, and the activity was calculated by measuring the content of glucose. The pET-30a plasmid was used as control

From *G. hirsutum* acc. TM-1 transcriptome data (Accession code PRJNA248163), *GhGLU19* homeologs (*Gh_A04G0109* and *Gh_D05G3612* in A- and D- subgenome from TM-1 genome sequences (NAU-NBI_V1.1), named as *GhGLU19A* and *GhGLU19D*, respectively) showed a preferential expression during the process of seed germination and early seedlings (Fig. 1A). The qRT-PCR analyses further confirmed that *GhGLU19* showed an upregulation expression during seed germination, with higher expression in imbibed seeds of 8, 12, 24 and 48 h (Fig. 1B), indicating that the expression of *GhGLU19* was significantly correlated with the water uptake process during imbibition stage of seed germination.

Sequence analysis showed that the ORF length of *GhGLU19A* was 1,410 bp with three exons and two introns (Fig. S1A). *GhGLU19A* encoded a peptide of 469 amino acids and shared 98% identity with GhGLU19D. GhGLU19 belonged to subfamily B with two conserved domains of Glycosyl hydrolase family 17 and the X8 (Fig. S1B). Multiple alignment revealed that the GhGLU19 was highly homologous with β-1,3-glucanase in other cotton species (Fig. S1C)*.* Phylogenetic analysis showed that GhGLU19 and its orthologs existed widely in different plants (Fig. S2). In vitro enzyme activity assay revealed that GhGLU19 was able to hydrolyze β-1,3-glucan (callose) to glucose (Fig. 1C).

### Down-regulation of *GhGLU19* increased seed germination percentage in transgenic cotton

To verify the function of *GhGLU19* during the process of seed germination, *GhGLU19A* overexpression and antisense vectors were constructed and transformed to *G. hirsutum* acc. W0, respectively (Fig. S3A and B). Transgenic plants were screened by PCR amplification of *GhGLU19* and kanamycin resistant selection (Fig. S3C and D). After self-crosses at T_1_ and T_2_ generation, two homozygous *GhGLU19*-overexpressing lines (*GhGLU19*-OEs) and two *GhGLU19*-suppressing transgenic cotton lines (*GhGLU19*-ASs) were selected at T_3_ generation, respectively. Quantitative real-time PCR confirmed that the expression of *GhGLU19* was significantly increased in seeds from *GhGLU19*-OEs and dominantly decreased in *GhGLU19*-ASs (Fig. S3E).

**Fig. 2:**
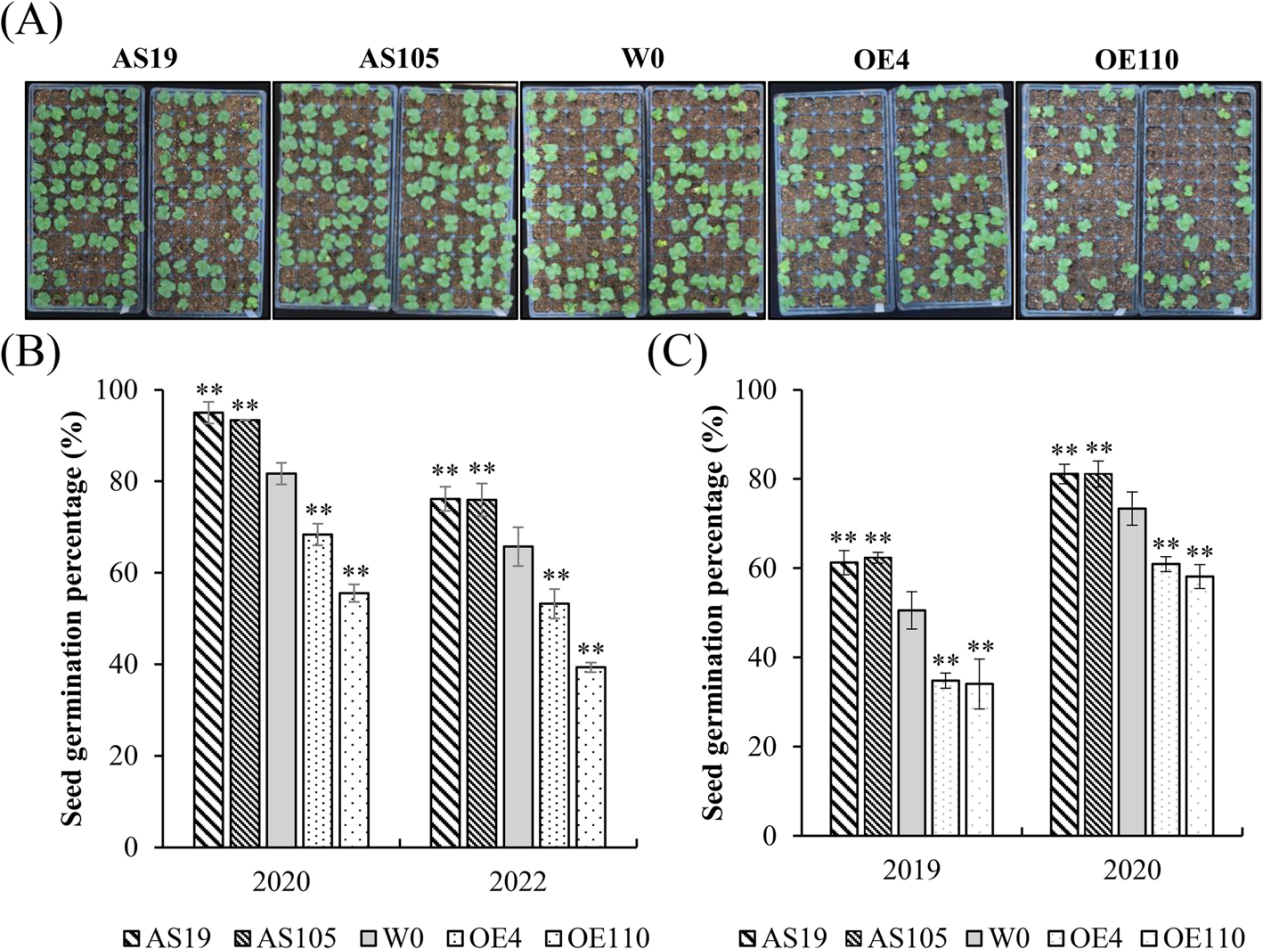
Suppression of *GhGLU19* increases seed germination percentage. **A**-**B** Seedling emergence phenotype (**A**) and seed germination percentage (**B**) of transgenic lines and W0 after sown 7 days at 25 °C growth chamber. Germination percentage as the means ± SD with three biological replicates. **C** Seed germination percentage of transgenic lines in field condition. Seeds of W0, *GhGLU19-*suppressing lines AS19 and AS105, *GhGLU19*-overexpressing lines OE4 and OE110 were planted in field, respectively, germination percentage were calculated after sown 3 weeks. Both in house and field germination assays were performed at least three independent biological replicates for each year with two year replications. Germination percentage as the means ± SD, ** *p* < 0.01

The effects of *GhGLU19*-OEs and *GhGLU19*-ASs on seed germination were examined in both lab and field conditions. In lab conditions, seeds from transgenic lines and control were planted in soils and germination rate were investigated in 2020 and 2022, respectively (Fig. 2A). In 2020, the germination percentage of AS19 and AS105 were 95.00 and 93.33%, significantly higher than W0 (81.67%), while the germination rate of OE4 and OE110 were 68.33 and 55.56%, respectively. Similar results were obtained in 2022 (Fig. 2B). In addition, the seed germination percentage was also investigated in the field condition. The results showed that *GhGLU19*-ASs had higher germination percentage than W0, while *GhGLU19*-OEs showed a significant decrease in germination. In 2019, the AS19 germination percentage (61.25%) was similar with AS105 (62.36%), both significantly higher than W0 (50.56%), while OE4 and OE110 showed the lower germination percentage with 34.81 and 34.07%, respectively, with the same tendency in 2020 (Fig. 2C). Both field trail and lab germination test indicated that suppression of *GhGLU19* promoted seeds germination percentage.

**Fig. 3:**
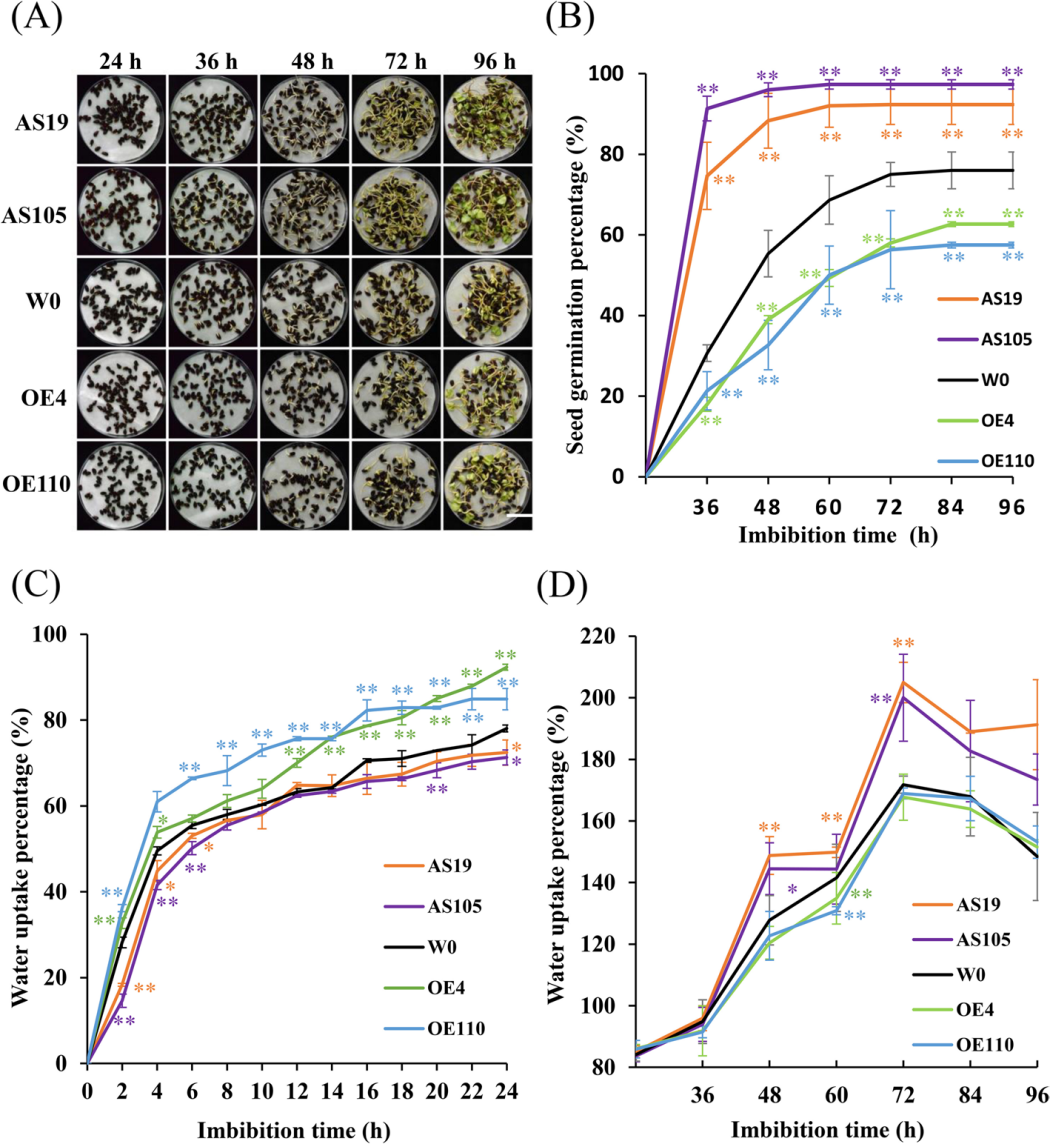
Seed germination and water absorption percentages in *GhGLU19*-overexpressing and *GhGLU19*-suppressing lines. **A** Photographic illustration of seed germination curve at 24, 36, 48, 72 and 96 h after imbibition. Scale bar, 3 cm. **B** Line chart of seed germination percentages in transgenic lines and W0 after 24, 36, 48, 60, 72, 84 and 96 h at 25 °C growth chamber. Germination percentage as the means ± SD with three biological replicates. **C**-**D** Water uptake time course of transgenic and wild-type seeds. Seeds from W0, *GhGLU19*-suppressing lines AS19 and AS105, *GhGLU19*-overexpressing lines OE4 and OE110, were sown in dishes at 25 °C, respectively. Percentage of water uptake was scaled by 2 h over one day. After 24 h, water uptake percentage was calculated each 12 h interval. Three independent biological replicates were analyzed. Water uptake percentage as the means ± SD, student’s *t*-test, * *p* < 0.05, ** *p* < 0.01

To illustrate the *GhGLU19* role involved in seed germination process, the germination assay was performed and seed germination process at different time point was further observed. The results showed that there were higher percentage of seeds germination (92.33 and 97.33%) in AS19 and AS105, and lower (62.67 and 57.50%) in OE4 and OE110, which was much higher and lower than that in W0 (76.00%), respectively (Fig. 3A-B). To illustrate the relationship between water uptake process and germination, water absorption percentage was investigated among *GhGLU19*-OEs, *GhGLU19*-ASs and the control. Water absorption of seeds from *GhGLU19*-OEs was initiated quickly and rapidly, after 24 h imbibition, reached at 92.33 and 84.89% in OE4 and OE110, respectively (Fig. 3C). However, the water absorption rate after 24 h was slowed down in both OE4 and OE110. Oppositely, the initial water uptake process was significantly slowed-down in *GhGLU19*-ASs, and an accelerated water absorption was observed after 24 h (Fig. 3D). Taken together, GhGlu19 is a regulator of seed water uptake process, especially functions on initial water absorption.

### *GhGLU19*-AS transgenic cottonseeds accumulated callose and showed altered imbibition curve

**Fig.4:**
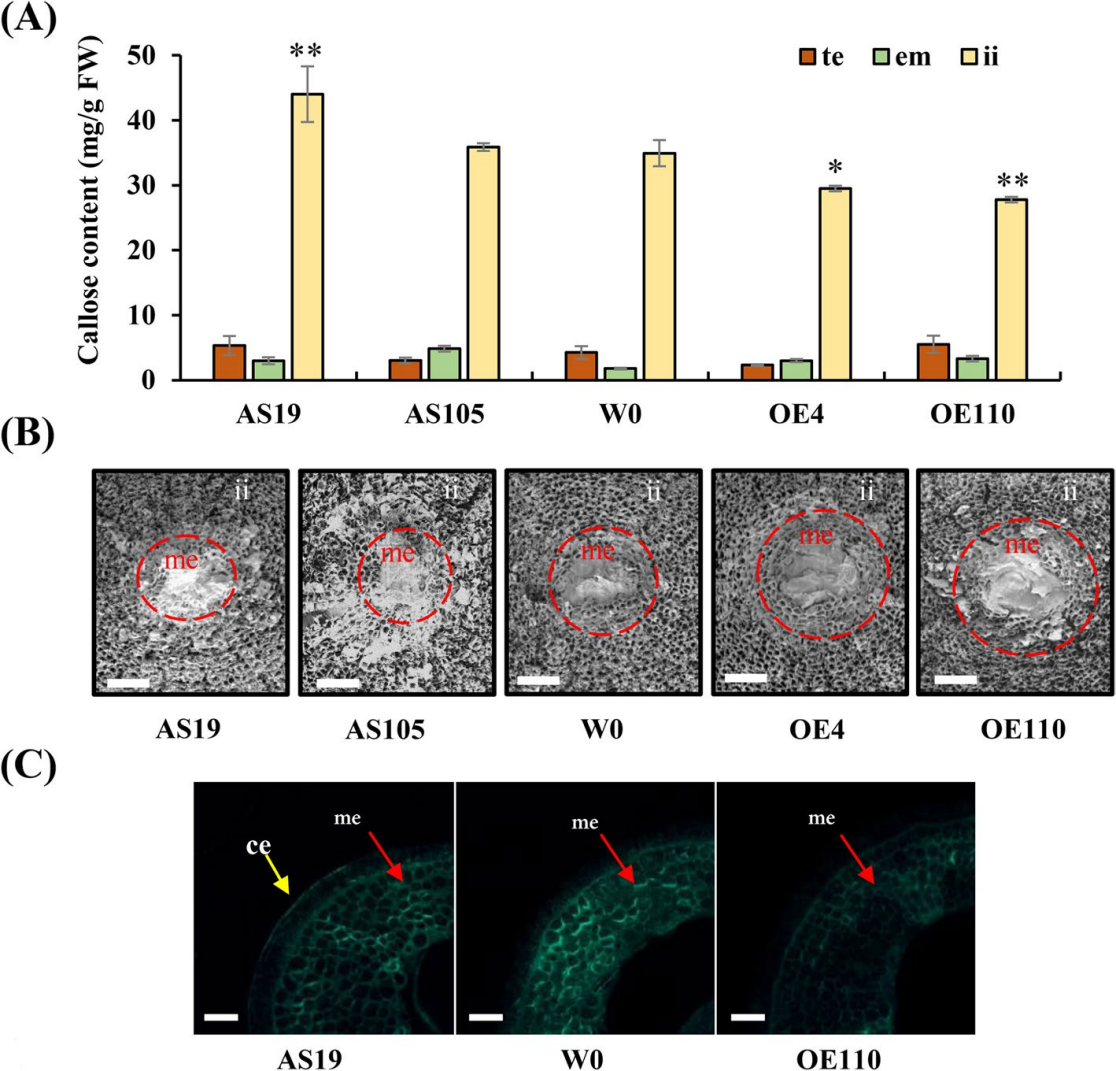
Callose accumulation was altered in *GhGLU19*-overexpressing and *GhGLU19*-suppressing lines. **A** Callose accumulation in embryos, seed coats and inner integuments were measured in 4 h imbibed seeds of *GhGLU19*-overexpressing and *GhGLU19*-suppressing lines. te, testa; em, embryo; ii, inner integument. Three independent biological replicates were analyzed. Student’s *t*-test, * *p* < 0.05, ** *p* < 0.01. **B** Callose deposition at micropylar region of different transgenic seeds. Scanning electron micrographs of micropyle showed that callose was highly accumulated in micropylar region of transgenic seeds of *GhGLU19*-suppressing lines. me, micropylar endosperm. Scale bar, 50 μm. **C** Callose detection on transection of micropyle of different transgenic seeds. Sections were stained with 0.05% aniline blue buffer, and pictured under UV light. ce, callose envelope. Scale bar, 500 μm

To determine how GhGLU19 affects callose accumulation in seeds, we measured the callose content in embryos, seed coats and ii of seeds from transgenic lines and wild-type. In seeds, callose was significantly enriched at ii. At 4 h imbibed seeds, callose content in ii from *GhGLU19*-ASs showed significant increase, with about 25% higher in AS19 than those in W0. Meanwhile, the callose content in *GhGLU19*-OEs showed significant decrease, which the amount in OE4 and OE110 were about 85 and 80% of those in wild-type seeds (Fig. 4A). These results indicated that suppression of *GhGLU19* increased callose accumulation in ii of seeds.

Histochemical staining of βGlu I promoter activity showed that the signals mainly localized in the inner testa, especially in micropylar region [1]. Scanning electron microscope observation of *GhGLU19*-ASs micropyle showed a dominant cap-like structure, which might result from increased callose accumulation in comparison with the control (Fig. 4B). To confirm callose accumulation at micropyle, ultrathin resin embedded sections from micropyle of imbibed seeds were further observed by aniline blue staining. In *GhGLU19*-AS lines, aniline blue signal was observed as an envelope at the margin of micropyle region. However, no margin callose layer was observed in wild-type or *GhGLU19*-OEs (Fig. 4C). These data indicated that GhGLU19 regulated callose deposition in seeds especially in ii and micropylar region.

### Glycolysis and pyruvate metabolism was affected in imbibition seeds of *GhGLU19* transgenic cotton lines

**Fig. 5:**
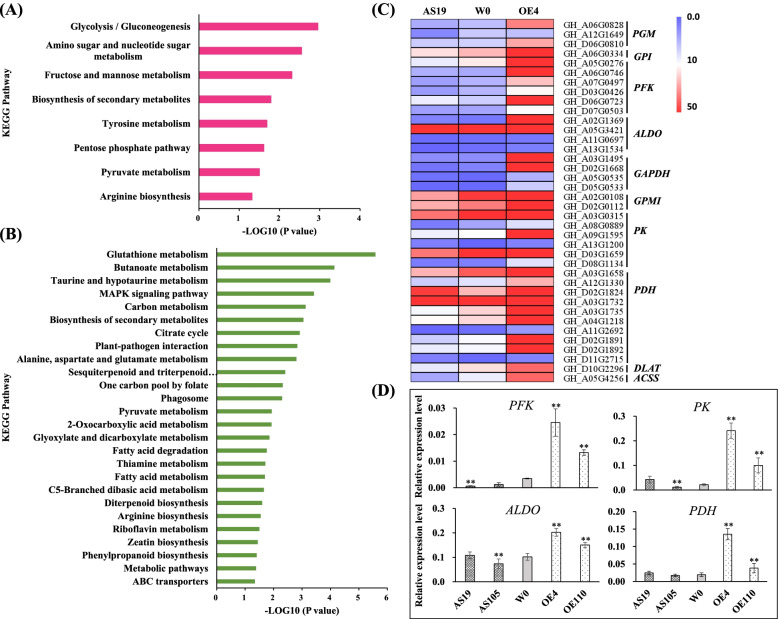
Glycolysis and pyruvate metabolism pathway was changed in early germination of transgenic plants. **A**-**B** KEGG pathway analysis of DEPs (**A**) and DEGs (**B**) from *GhGLU19*-overexpressing line OE4 compared to W0 indicated that glycolysis and pyruvate metabolism were highly enriched. **C** Genes related to glycolysis and pyruvate metabolism were highly expressed in OE4. These genes were selected from KEGG map (ko00620 and ko00010) [21]. **D** Expression clarification of glycolysis and pyruvate metabolism related genes in transgenic and W0 seeds by qRT-PCR. Genes related to glycolysis and pyruvate metabolism were involved in *PFK*, *ALDO*, *PK* and *PDH*. Their expression levels were significantly evaluated in 8 h imbibed seeds of *GhGLU19*-overexpressing transgenic lines compared to W0. Relative expression was calculated using *Histone3* (*AF024716*) as an internal reference. Three independent biological replicates were analyzed with three technical repeats. Vertical bars represent means ± SD. * *p* < 0.05. ** *p* < 0.01

To investigate whether metabolic activities might be altered during seed germination of the transgenic lines, an iTRAQ-based proteomic profiling analysis was performed to identify the differences in protein composition of imbibed seeds from AS19, OE4 and W0. A total of 1881 unique proteins were identified. Of them, 79 (39 up-regulated and 40 down-regulated) and 143 (63 up-regulated and 70 down-regulated) DEPs were further detected in comparison of AS19 vs W0 and OE4 vs W0, respectively (Dataset S1, S2). Pathway enrichment analysis of DEPs in comparison group of OE4 vs W0 showed that pathways related to glycolysis, biosynthesis of secondary metabolites, amino acid metabolism, and pyruvate metabolism were highly enriched (Fig. 5A). Oppositely, several key enzymes involved in glycolysis, pyruvate metabolism, and ABA biosynthesis were decreased in comparison group of AS19 vs W0 (Dataset S1).

Based on 2-fold difference in transcript levels (p.adj < 0.05; log_2_FC > 1 or log_2_FC < -1), a total of 523 DEGs were identified in comparison of AS19 vs W0 (Dataset S3), and 3732 DEGs in comparison of OE110 vs W0 (Dataset S4). KEGG enrichment analysis showed that the genes related to pyruvate metabolism, biosynthesis of secondary metabolites, and amino acid metabolism were differentially activated in comparison group of OE110 vs W0 (Fig. 5B), and most of them were significantly decreased in comparison group of AS19 vs W0 (Fig. 5C), which was in an agreement with the result of proteome analysis. Furthermore, we analyzed the transcripts of genes involved in glycolysis and pyruvate metabolism, including *phosphoglucomutase* (*PGM*), *glucose-6-phosphate isomerase* (*GPI*), *6-phosphofructokinase* (*PFK*), *fructose bisphosphate aldolase* (*ALDO*), *glyceraldehyde 3-phosphate dehydrogenase* (*GAPDH*), *2,3-bisphosphoglycerate-independent phosphoglycerate mutase* (*PGMI*), *pyruvate kinase* (*PK*), *pyruvate dehydrogenase* (*PDH*), *dihydrolipoamide acetyltransferase* (*DLAT*) and *acetyl-CoA synthetase* (*ACSS*), in imbibed seeds of AS19, OE110 and W0, respectively. The result showed that the transcript levels of these genes were slightly decreased in AS19 but dominantly increased in OE110, compared to W0 (Fig. 5C). Four genes encoding key enzymes in glycolysis and pyruvate metabolism, *PFK*, *ALDO*, *PK* and *PDH*, were further selected to detect their expression in early germinating seeds of transgenic lines (Fig. 5D). qRT-PCR showed that compared with W0, the expression of the four genes were significantly increased in *GhGLU19*-overexpressing lines, while were decreased in *GhGLU19*-ASs. These results indicated that glycolysis and pyruvate metabolism were changed in early germination of transgenic seeds and affected seed germination.

### Endogenous ABA content and ABA signaling was altered in imbibition seeds of *GhGLU19* transgenic cotton lines

**Fig. 6:**
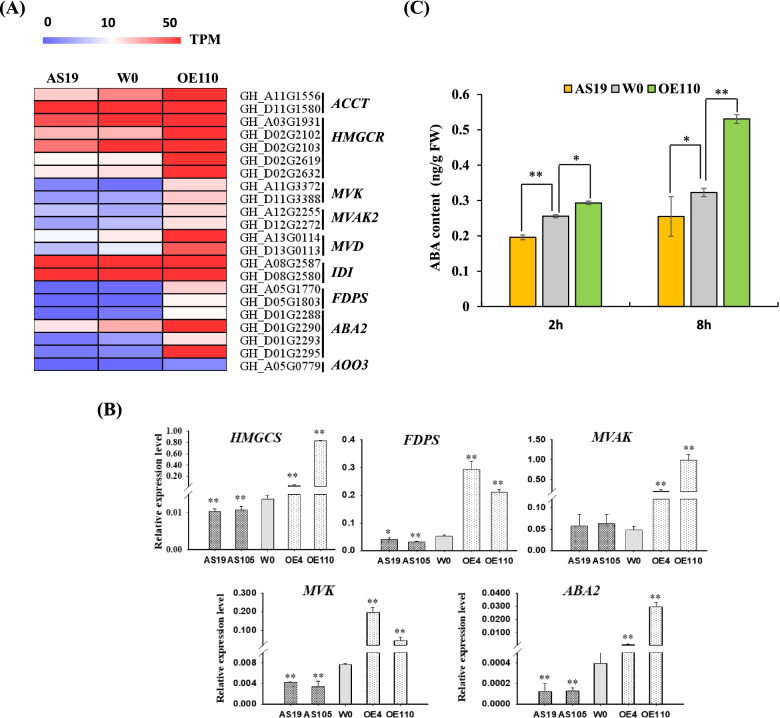
ABA biosynthesis was deregulated in GhGLU19-Ass. **A** Expression heatmap of genes related to MVA and ABA biosynthesis. **B** Transcript levels of MVA pathway and ABA biosynthesis related genes in transgenic lines and W0 by qRT-PCR analysis. Genes encoding key rate-limiting enzymes in MVA and ABA biosynthesis were involved in *HMGCS*, *MVK*, *MVAK*, *FDPS* and *ABA2*. Their expression levels were significantly evaluated in 8 h imbibed seeds of *GhGLU19*-overexpressing transgenic lines compared to W0. Relative expression was calculated using *Histone3* as an internal reference. Three independent biological replicates were analyzed with three technical repeats. Vertical bars represent means ± SD. * *p* < 0.05. ** *p* < 0.01. **C** Endogenous ABA content of imbibed seeds at 2 h and 8 h were measured by high performance liquid chromatography with tandem mass spectrometry among transgenic lines and W0. Three independent biological replicates were analyzed. Vertical bars represent means ± SD. * *p* < 0.05. ** *p* < 0.01

KEGG enrichment showed that the accumulation of proteins was also involved in ABA biosynthesis via MVA pathway, including phosphomevalonate kinase (MVAK), hydroxymethylglutaryl-CoA synthase (HMGCS), xanthoxin dehydrogenase (ABA2) (Dataset S2). Meanwhile, DEGs related to biosynthesis of secondary metabolites mainly focused on terpenoid backbone biosynthesis by MVA and ABA biosynthesis pathway, which was supported by the enhanced transcripts of *Acetyl-CoA C-acetyltransferase* (*ACCT*), *hydroxymethylglutaryl-CoA reductase* (*HMGCR*), *mevalonate kinase* (*MVK*), *MVAK*, *diphosphomevalonate decarboxylase* (*MVD*), *isopentenyl-diphosphate Delta-isomerase* (*IDI*), *farnesyl diphosphate synthase* (*FDPS*), *ABA2*, and *abscisic-aldehyde oxidase* (*AAO3*) (Fig. 6A).

**Fig. 7:**
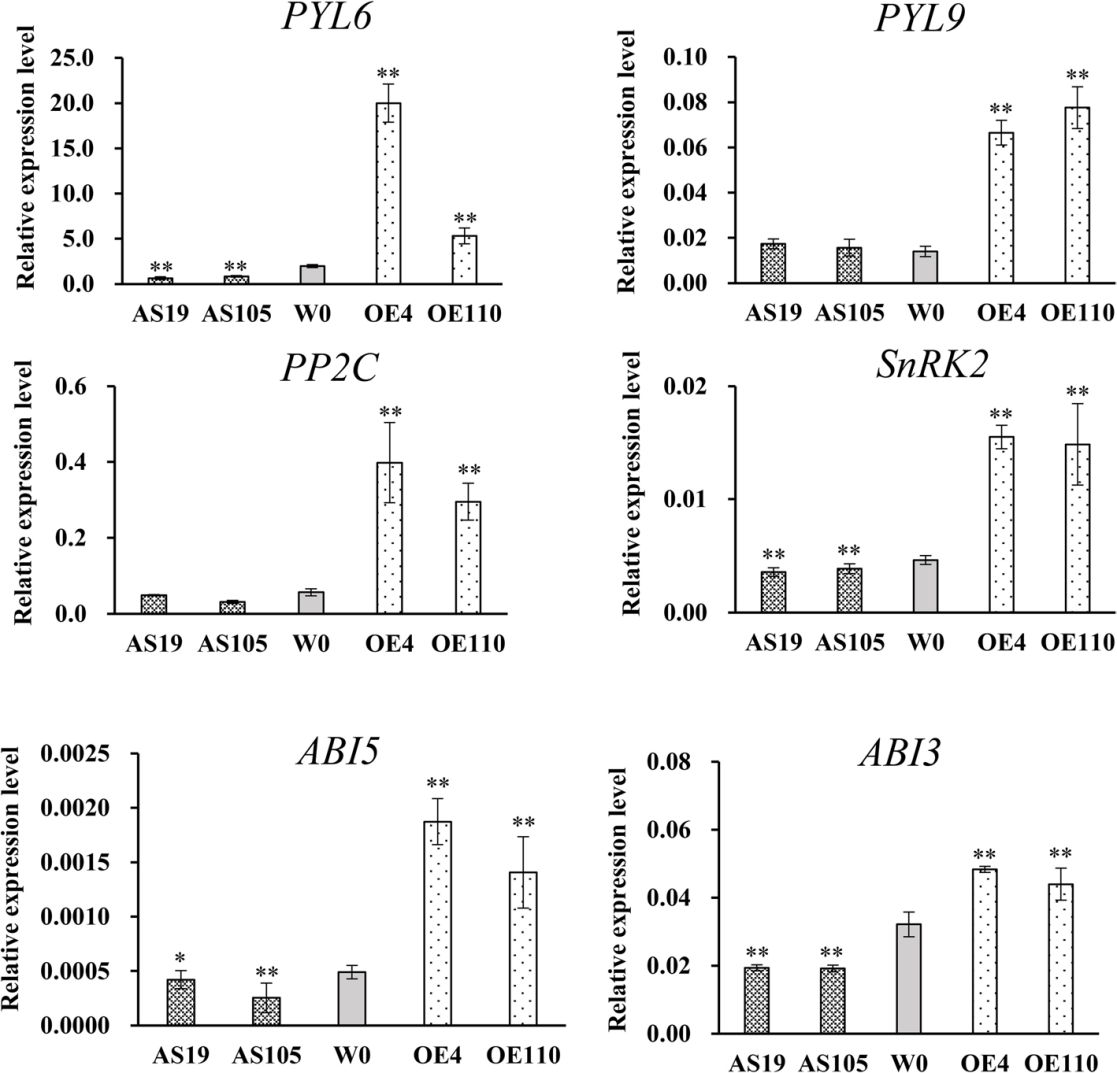
Expression analysis of ABA signaling genes in transgenic lines and wild type. qRT-PCR analysis of ABA signaling related genes including ABA receptors (*PYL6* and *PYL9*), *PP2C*, *SnRK2*, *ABI3* and *ABI5*, was performed using 8 h imbibed seeds from transgenic lines and W0. Relative expression was calculated using *Histone3* as an internal reference. Three independent biological replicates were analyzed with three technical repeats. Vertical bars represent means ± SD. * *p* < 0.05. ** *p* < 0.01

HMGCS, MVK, MVAK, FDPS and ABA2 are essential enzymes of ABA biosynthesis. The qRT-PCR analysis further confirmed that transcription of these genes was significantly downregulated in *GhGLU19*-ASs compared with the W0. By contrast, all these genes were substantially elevated in *GhGLU19*-overexpressing imbibition seeds (Fig. 6B). Further, ABA content was detected by HPLC–MS/MS in AS19, OE110 and W0. In 2 h and 8 h imbibition seeds, ABA accumulation in OE110 was significantly higher than WT, while it was dramatically lower in AS19 (Fig. 6C). We also detected the transcript levels of genes involved in ABA signaling pathway. The qRT-PCR results showed that the transcript levels of six genes, including *pyrabactin resistance 1-like 6* (*PYL6*), *PYL9*, *abscisic acid insensitive 3* (*ABI3*), *ABI5*, *protein phosphatase 2C* (*PP2C*) and *sucrose nonfermenting 1-related protein kinase 2* (*SnRK2*), were much higher in OE lines and lower in ASs compared with that in W0 (Fig. 7). These results indicated that the increased seed germination in *GhGLU19*-ASs was partially dependent to decreased ABA content and ABA signaling pathway.

## Discussion

### Physiological roles of GhGLU19 during seed germination

Seeds from many eudicot plants including cotton had a callose layer at the interface of the maternal seed coat and the endosperm or embryo. Endosperm exposed as a cap-like structure that covers radicle tip at the micropyle, which is necessary to maintain water impermeability [12]. The micropylar endosperm is an obstacle to radicle emergence, whose weakening is thought as a prerequisite for completion of seed germination in many dicotyledons. It has been reported that several cell wall genes are expressed exclusively in the endosperm cap, which suggests their activities in endosperm weakening [22]. Wu et al*.* [5] reported that class I isoforms of β-1,3-glucanase was expressed specifically in the endosperm caps of tomato seeds, but it is unlikely that this enzyme was directly involved in cell wall modification or tissue weakening during germination. It is worth to discuss that β-1,3-glucanase does other possible roles during germination. In our study, expression of *GhGLU19* was induced exclusively after seed imbibition, indicating that *GhGLU19* is involved in regulating seed germination (Fig. 1B). We also found that seeds of *GhGLU19*-ASs had over-accumulation of callose in inner integument of seeds, including micropylar region. In previous reports, uptake of water by a dry seed follows a classic triphasic model, with a rapid initial uptake (phase I) followed by a plateau phase (phase II), and a final rapid water uptake period [14]. Physically, certain water uptake time (phase I) is necessary, because imbibition metabolism must be initiated to protect their cytoplasmic components and the recovery from seed structural damage caused by maturation drying and oxidation [15]. In our study, a slowed-down water uptake process and higher seed germination percentage were detected in GhGLU19-ASs than that in W0 and GhGLU19-OEs, which may be related to the prolonged phase I period for repair and reestablishment of metabolic activity in mitochondria. Furthermore, the seeds of GhGLU19-OEs showed a decreased callose accumulation and the lower seed germination percentage (Figs. 2 and 3). This suggest that certain amount of callose layer be important as a channel charging for water influx into seeds during seed imbibition, which ensured the cell components to be recovered into a normal structure after water uptake initiated.

### Downregulation of *GhGLU19* causes the decrease of glycolysis and pyruvate metabolisms in imbibed seeds

The poor timing of seed germination can severely limit seedling survival [23]. Both proteomic profiling and transcriptome analysis showed that expression of proteins regulating glycolysis and pyruvate metabolism were significantly activated in the seeds of *GhGLU19*-OEs during early germination stage (Fig. 5). It has been reported that mitochondria provide metabolic intermediates, reductant and ATP essential for biosynthesis and cell expansion. However, mitochondria in the dry seed have a poorly developed internal membrane structure with few cristae, which will be recovered into a normal structure after water uptake initiated [24]. Glycolysis is an anaerobic metabolic pathway, and it is also the first step in cellular respiration. In glycolysis, one molecule of glucose and 2 ATP molecules are consumed, with the production of 4 ATP, 2 NADH, and 2 pyruvates [25]. The pyruvate, the product of glycolysis, is utilized in the biosynthesis of numerous compounds such as secondary metabolites, amino acids, nucleic acids, and fatty acids. Therefore, glycolysis and pyruvate metabolism are particularly important in actively growing autotrophic tissues, such as germinating seeds [26]. However, in anaerobic environment, pyruvate produces lactate or ethanol via respiration [27]. Both these two products are not conducive to growth, which leads to a considerable carbon depletion and toxicity during germination [28]. In this study, qRT-PCR analysis revealed that the transcript levels of genes related to glycolysis and pyruvate metabolism were significantly elevated in seeds of *GhGLU19*-OEs (Fig. 5), for example, PFK, the ‘gatekeeper’ of glycolysis, which catalyze the committed step of the glycolytic pathway by converting fructose-6-phosphate to fructose-1,6-bisphosphate [29]. Meanwhile, the relatively fast water uptake process in *GhGLU19*-OEs led to pyruvate metabolism pathway to be altered in the anaerobic environment, and produced the harmful lactate or ethanol during seed germination. From transcriptome data, we also found that genes encoding alcohol dehydrogenase and lactate dehydrogenase, which catalyzed pyruvate to ethanol or lactate without the consumption of O_2_, showed dominant higher expression in *GhGLU19*-OEs compared to W0 and *GhGLU19*-ASs (Fig. S4). Taken together, the decrease on expression of genes related to glycolysis and pyruvate metabolism and slowed water uptake process in early seed imbibition, which led to a lower but extended glycolysis and pyruvate pathway influx, brought about much energy supply for following respiration in seed germination and seedling growth.

### *GhGLU19* is involved in regulating ABA homeostasis during germination

During germination, seeds undergo a cascade of biochemical and mechanical events regulated by internal and external stimuli [30]. Germination starts with water uptake and requires both activation of embryo growth potential and weakening of a mechanical constraint such as testa or endosperm, both of which appear to be regulated by endogenous hormones [31]. Among these hormones, ABA is important for the induction and maintenance of seed dormancy and inhibits seed germination [32]. Both embryo and endosperm can synthesize ABA and release ABA to regulate embryonic growth [33, 34]. In cytosol, Ac-CoA is catalyzed by HMGCS to form HMG-CoA, the initiated substrate for MVA pathway to produce isopentenyl diphosphate (IPP) [35]. IPP is condensed to form GPP (C10, monoterpene backbone), FPP (C15, sesquiterpene backbone), and GGDP (C20, diterpene backbone) [36]. ABA is a C15 weak acid, synthesized from FPP, that was catalyzed by NCEDs, ABA2, ABA3 and AAO3 [37, 38]. In transgenic poplar, overexpression of HMGCR resulted in the increased ABA content by accelerated gene expression of MVA pathway and MEP pathway [39]. In this study, iTRAQ assay and transcriptome data revealed that the expression of these genes related to ABA biosynthesis and signaling pathway were down-regulated in *GhGLU19*-ASs, oppositely, those were highly expressed in *GhGLU19*-OEs. Furthermore, ABA content in OE110 was significantly higher than WT, while it was dramatically lower in AS19 (Fig. 6C). These findings demonstrate that ABA biosynthesis were oppositely regulated in *GhGLU19*-ASs and *GhGLU19*-OEs.

ABA signaling pathway also plays an important role on germination, which primarily acts by a cascade that comprises ABA receptors (encoded by PYRABACTIN RESISTANCE1 [PYR1]/ PYL 1–13), PP2Cs and SnRKs [40, 41]. Overexpression of rice PYLs showed improvement on drought resistance and ABA hypersensitivity in inhibition of seed germination [42]. PP2Cs also have essential roles in the release of seed dormancy epistatic to DELAY OF GERMINATION 1 [43]. SnRK2.2, SnRK2.3 and SnRK2.6 were mainly expressed during seed development and germination. These SnRK2s were involved in ABA signaling to regulate gene expression through the phosphorylation of transcription factors (TFs), such as ABI3 and ABI5 [44]. ABI3 is a major ABA responsive TF in ABA signaling, that plays a crucial role in seed germination [45]. Previous studies demonstrated that ABI5 acted downstream of ABI3 for controlling germination and seedling growth [46]. In our study, the transcript levels of genes related to ABA signaling components including *PYL6*, *PYL9*, *ABI3*, *ABI5*, *PP2Cs* and *SnRK2s* were decreased in *GhGLU19*-ASs, implying that suppression of *GhGLU19* showed the increased seed germination, partially due to decreased ABA level and down-regulated ABA signal.

Gibberellin (GA) has been identified as an essential plant hormone for seed germination. Bioactive GAs are diterpene acids, which are catalyzed from GGDP. The biosynthesis of GAs in higher plants can be divided into three stages: biosynthesis of ent-kaurene from GGDP by ent-copalyl diphosphate synthase and ent-kaurene synthase; conversion of ent-kaurene to GA12 via cytochrome P450 monooxygenases, and formation of C20- and C19-GAs in the cytoplasm [47]. From the transcriptome data, we found that the expression levels of gibberellin biosynthesis and signaling genes were not influenced by modulation of *GhGLU19* (Fig. S5). The transcripts of *ent-kaurene oxidase*, *GA 3-oxidase1* and *GA 20-oxidase2* are observed in the cortex and endodermis in the embryo axis, and suggesting that the synthesis of bioactive GAs from ent-kaurene occurs in the cortex and endodermis [48]. However, *GhGLU19*-modulated lines altered callose accumulation in inner integument of seeds but not in embryo and testa (Fig. 4). We speculated that *GhGLU19*-ASs with over-accumulated callose in inner integument, which affects ABA biosynthesis in endosperm and regulates seed germination.

## Conclusions

Compared to wild type, *GhGLU19*-suppressing and *GhGLU19*-overexpressing transgenic cotton lines showed the higher and lower seed germination percentage, respectively. Suppression and overexpression of *GhGLU19* showed a dominant increase and decrease of callose content in inner integument of seeds, respectively, which led to the relatively slow influx of water in *GhGLU19*-suppressing lines (ASs) and fast in *GhGLU19-*overexpressing lines (OEs) in the process of seed germination. Proteomic data and transcriptome analysis illustrated that the expression of genes involved in glycolysis and pyruvate metabolism were decreased in ASs, however, significantly increased in OEs. In addition, the fine-tuned callose content and following physiological activities might cause the decrease in expression of genes related to ABA biosynthesis and endogenous ABA level in ASs. The transcript levels of ABA signaling related genes were also decreased in ASs, which was opposite in OEs. Taken together, suppression of *GhGLU19* plays important roles in callose accumulation, glycolysis and pyruvate metabolism, and ABA biosynthesis and signaling pathway in imbibition seeds, which promotes seed germination. Future studies should focus on elucidating the precise mechanisms involved in how suppression of *GhGLU19* functions in plant growth, especially in seed development and germination.

## Methods

### Plant materials, vector construction and plant transformation

*G. hirsutum* acc. W0 was used as the wild-type (WT) control and genetic transformation receptor. DNA and RNA were extracted from one-month old cotton plants using Super Plant Genomic DNA kit and RNAprep Pure Plant Kit (TIANGEN, Beijing, China). To generate *GhGLU19* overexpression and antisense transgenic cotton lines, the full-length coding sequence of *GhGLU19* was amplified using primers OE-GhGLU19-F/R. The fragments were then inserted into *Xba* I and *Bam*H I sites of pBI121 vector through standard cloning procedures [49]. pBI121 is a binary vector, with kanamycin-resistance for plant selection, that expression of gene of interest was controlled by *Cauliflower mosaic virus 35S* promoter. For the antisense construct, the ORF sequence was reversely inserted into pBI121 vector following the same cloning procedures.

The constructs were introduced into W0 through *Agrobacterium*-mediated transformation following the method described previously [50]. Transgenic lines were self-crossed continuously for obtaining the homozygous lines at T_3_ generation. Both kanamycin selection and PCR amplification were used for determining transgenic homozygosity in each generation. All necessary permits for planting and investigating of transgenic materials and receptor were obtained from Nanjing Agricultural University, Jiangsu Province, China. The gene expression level was detected by quantitative real-time PCR (qRT-PCR) using *tubulin* (*KF555285.1*) as a reference gene. The primers are shown in Table S1.

### Germination assays

Seeds were delinted using 100% sulfuric acid and washed thoroughly at least three times by using sterilized ddH_2_O. For in house germination assay, seeds of control groups and transgenic lines were planted in soils and grown under the room temperature: 25 °C/23 °C day/night and 60% relative humidity with 16 h /8 h light/dark photoperiod. Germinated seedlings were counted and examined at 7 days after sowing. To evaluate germination of transgenic cotton and control in the field, 30 seeds were planted for each row and each line was planted for 4 rows. About three weeks after germination, seedling number were counted and calculated for germination rate. Both in house and field germination assays were performed at least three independent biological replicates for each year with two year replications.

For water absorption and germination time curve, 2 layers of filter paper were laid in a germination box. In each box, one hundred seeds were put and 40 mL water was added. Three independent biological replicates were performed. Germination boxes were put in an incubator to cultivate under the temperature: 25 °C/23 °C day/night and 80% relative humidity with 16 h /8 h light/dark photoperiod. Seeds was weighted at a 2 h interval until 24 h. After 24 h, the seed germination percentage was count at a 12 h interval time points, and the water absorption value also measured at the same time point.

### Quantitative real-time PCR

Total RNA was isolated from 100 mg leaf tissue of wild-type and transgenic cotton lines using CTAB-acidic phenolic method. Isolated total RNAs (0.5 μg) were reverse‐transcribed into cDNA using the HiScript Q RT SuperMix for qPCR (+ gDNA wiper) (Vazyme, Nanjing, China). Gene-specific primers for qRT-PCR analysis were designed using Beacon Designer 7.0. Real-time PCRs were performed in a Roche 480 PCR system using AceQ SYBR Green Master (Low Rox Premixed) (Vazyme), with three technical replicates for each biological sample. The expression data were evaluated using the comparative cycle threshold method described previously [51]. Data analyses were presented as means ± standard deviation (SD) of three biological replicates. Detailed information of primers for qRT-PCR was shown in Table S1.

### Callose extraction and detection

Callose contents in 2 h and 4 h imbibed seeds were quantified by using spectrophotometric methods, that aniline blue staining was widely assumed to indicate the presence of 1,3-β-glucans [52]. Seeds were dissected, and embryo, testa and ii, separately. The samples (100 mg) were ground in liquid nitrogen and disintegrated in 3 mL NaOH. The suspension was incubated at 80 °C for 15 min to solubilize the callose and then centrifuged (5 min, 380 × g). About 200 μL of supernatant was then removed to a new 2 mL tube, and mixed with 400 μL of 0.1% (w/v) aniline blue. pH of solution was adjusted using 210 μL HCl and 590 μL glycine–NaOH buffer (pH 9.5). The solution was incubated at 50 °C for 20 min and another 30 min at room temperature later. Fluorescence was read on an Inifinitc M200 (Tecan) spectrofluorometer (excitation 400 nm, emission 510 nm, and slit 10 nm). Calibration curves were established using a freshly prepared solution of the 1,3-β-glucan laminarin (L9634, Sigma) in NaOH buffer. The standard curve was calibrated by 0, 0.1, 1, 2.5, 5 and 10 μg μL^−1^. Using the calibration curve, amounts of callose in cottonseeds were estimated as laminarin-equivalents. Data analyses were presented as means ± SD of three biological replicates.

### *In vitr*o expression of GhGLU19 and enzyme activity assay

The predicted signal peptide (amino acids 1–23) was removed from *GhGLU19* open reading frame sequence, and the remaining sequences were cloned into pET-30a under control of a strong bacteriophage T7 transcription and translation signals. The recombinant *Escherichia coli* strain BL21 (DE3) carrying constructs (the pET-30a plasmid as control) were cultivated and induced with 0.5 mg L^−1^ Isopropyl β-D-thiogalactopyranoside for protein expression.

Enzyme activity of GhGLU19 was detected by the dinitro salicylic acid (DNS) assay, in which glucanase activity was calculated by measuring the rate of increase in the absorbance at 530 nm due to the formation of glucose, as described previously [53]. The enzyme assay was performed by adding 0.1 mL of crude enzyme solution to 0.4 mL of 0.1% (w/v) laminarin solution. The reaction mixture was incubated at 40 °C for 30 min, followed by incubation in boiling water for 5 min, then added 0.5 mL of DNS solution and incubated at boiling water for 5 min. The glucose was detected using a spectrophotometric method as an indicator of β-glucanase activity. Data analyses were presented as means ± SD of three biological replicates.

### ABA extraction and detection

Endogenous ABA was extracted in imbibed cottonseeds following the previous report [54]. After grinding under liquid N_2_ to fine powder, 500 mg was used for extraction. Extraction of the free analytes was carried out with 5 mL isopropanol/hydrochloric acid extraction buffer and the internal standard Abscisic Acid-d6 (ChemeGen). Samples were incubated at 4 ºC for 30 min after adding dichloromethane. After centrifugation at 13,000 × g, the organic supernatant was transferred to a new tube. After drying in a gentle stream of N_2_, the pellets were dissolved in 400 μL methanol. Determination of the analytes in 1 mL injected volume was performed by HPLC–MS/MS.

ABA was measured with a HPLC-ESI-QTRAP MS instrument (6500 Qtrap, Applied Biosystems / MDS SCIEX, Concord, ON) fitted with Poroshell 120 SB-C18 (2.7 µm, 2.1 × 150 mm, Agilent, Portland, OR). The column temperature was kept at 30 °C. A binary mobile phase system consisting of 0.1% (v/v) formic acid (A) and methanol (B) under a gradient condition [0–1 min, 20% (B), 1–9 min, 20% (B) to 80% (B), 9–10 min, 80% (B) and 10–15 min, 80% (B) to 20% (B)] with a flow rate of 0.3 mL min^−1^ was used. Each injection volume was 2 μL. Products were further quantified in multiple-reaction-monitoring mode for ABA detection. Electrospray ionization was performed in the negative ionization mode, curtain gas of 25.0 psi, collision gas medium, 4500 V for ion spray voltage, source temperature at 400 °C, nebulize gas of 65 psi and desolution gas of 70 psi.

### iTRAQ assay

Total protein of 8 h imbibed seeds was extracted using phenol-based method and the concentrations were measured with bovine serum albumin. Next, 100 μg protein was used for iTRAQ labelling proteomics analysis [55]. Protein digestion and labelling were performed according to the manufacturer's instructions (AB SCIEX, Concord, ON). Data acquisition was performed with a TripleTOF 5600 mass spectrometer (MS) (AB SCIEX, Concord, ON) fitted with a Nanospray III source (AB SCIEX, Concord, ON). Data were acquired on the parameters as previously reported [55]. The original mass data were processed with ProteinPilot 5.0 (AB SCIEX, Concord, ON) and searched against the Uniprot protein database (http://www.uniprot.org) and *G. hirsutum* TM-1 protein FASTA (https://www.cottongen.org/) [56]. For identification of reliable protein, the following filters were used: significance threshold *P*-value < 0.05 (with 95% confidence) and for which at least one unique peptide was detected. The quantitative protein ratios were weighted and normalized to the median ratio. The significantly differentially expressed proteins (DEPs) were quantified in two biological replicates, along with a Fisher’s combined probability of < 0.05 and a fold change ± 1.5.

The Kyoto Encyclopedia of Genes and Genomes database (KEGG; http://www.genome.jp/kegg/pathway.html) was used to identify DEPs involved in biological, metabolic and signal transduction pathways. Fisher’s exact test was applied to compare the distribution of each KEGG pathway in the target protein cluster and the overall protein cluster. The background was constituted by the whole annotated gene sequence of *G. hirsutum* acc. TM-1 genome (ZJU-improved_v2.1_a1). *P* < 0.05 were considered statistically significant using a two-tailed Fisher’s exact test.

### Transcriptome sequencing

*GhGLU19* transgenic and W0 seeds were sown on two-layer filter paper with 30 mL water. After 8 h imbibition, seeds were collected and ground for total RNA extraction. RNA-seq (NovaSeq6000 platform) was performed to compare the transcript patterns of *GhGLU19* transgenic lines and W0. The raw data were processed quality control by using an NGS QC toolkit. Hisat2 was used for mapping the processed reads to the *Gossypium hirsutum* acc. TM-1 genome (ZJU-improved_v2.1_a1) with default parameters [57]. Finally, the transcript abundance was determined using HTSeq package, and differential expression analysis was accomplished with a *P*-adjusted value < 0.05 cutoff (q-value) and a fold change of > 2 by edgeR [58]. Differentially expressed genes (DEGs) were analyzed by KEGG pathway enrichment. All samples contained three biological repeats. Mev 4.9 was used for a heatmap illustration of the expression patterns, based on transcripts per million (TPM) values [59].

### Seed fixation, embedding and sectioning

Seed fixation, embedding and sectioning were conducted according to Pugh et al. [60]. After 2 h imbibition, seed was cut from central section, and testa was removed carefully without contacting inner seed coat. Immediately, dissected seed samples were fixed in a 20% step-graded ethanol series from 30 to 100% at 2 h intervals. The 100% ethanol was treated with 5% (w/v) molecular sieve for water absorption. Samples were then stored in 100% ethanol at 4 °C, with ethanol changes every day, for 3 days. Samples were infiltrated into LR White Resin (ProSci Tech, Inc., Qld, Australia) 20% step-graded series from 30 to 70% with a 2 h interval. Infiltration into resin was performed by 10% step-graded series from 70 to 100%. The samples were treated with resin changes every 3 days for six times. Infiltrated samples were transferred to fresh resin in an appropriate mold and baked at 60 °C for 24 h. Thin Sects. (2 μm thick) were cut using an ultramicrotome (UC7, Leica, Germany) with a glass knife. The sections were placed onto glass slides and fixed for staining.

### Aniline blue staining

Aniline blue staining was performed using aniline blue (A113197, Aladdin) as described by Vate´n et al. [61]. A stock solution (10 mg mL^−1^ in ddH_2_O) was diluted 1:10 ratio with PBS (pH 9.5). Sections were incubated in the staining solution for 1 h at room temperature. The signal of aniline blue was imaged in PBS solution on an Olympus BX53 microscope (Japan) using a UV laser.

## Supplementary Information

**Additional file 1: Figure S1.** Structure and characterization of GhGLU19.

**Additional file 2: Figure S2.** Phylogenetic analysis of GLUs from G. hirsutum and other plants.

**Additional file 3: Figure S3.** Identification of GhGLU19-overexpressing and GhGLU19-suppressing transgenic cotton lines.

**Additional file 4: Data S1.** Information on 79 differential expression proteins (unsed >1.3 and a fold change>1.5) by comparative analysis between AS19 and WT.

**Additional file 5: Data S2. **Information on 143 differential expression proteins (unsed >1.3 and a fold change>1.5) by comparative analysis between OE110 and WT.

**Additional file 6: Data S3. **Information on 523 differential expression genes (*p* value<0.05 and a fold change>2) by transcriptome comparative analysis between AS19 and WT.

**Additional file 7: Data S4. **Information on 3732 differential expression genes (*p* value<0.05 and a fold change>2) by transcriptome comparative analysis between OE110 and WT.

**Additional file 8: Figure S4**. Expression heat map of genes encoding alcohol dehydrogenase and lactate dehydrogenase in transgenic and control imbibed seeds.

**Additional file 9: Figure S5**. Expression heat map of gibberellin biosynthesis and signaling genes in transgenic and control imbibed seeds.

**Additional file 10: Table S1.** Primers used in this study.

## Data Availability

The genomic database of different cotton species was downloaded from the CottonGen database, and links to all databases as follows. *G. arboreum*: https://www.cottongen.org/species/Gossypium_arboreum/bgi-A2_genome_v2.0; *G. barbadense*: https://www.cottongen.org/species/Gossypium_barbadense/nbi-AD2_genome_v1.0; *G.hirsutum*: https://www.cottongen.org/species/Gossypium_hirsutum/nbi-AD1_genome_v1.1; *G. mustelinum*: https://www.cottongen.org/species/Gossypium_mustelinum/JGI-AD4_v1.1; *G. raimondii*: https://www.cottongen.org/species/Gossypium_raimondii/jgi_genome_221; *G. tomentosum*: https://www.cottongen.org/species/Gossypium_tomentosum/HGS-AD3_v1.1; The genome sequence data of 15 plant species were downloaded from the public databases, and links to all databases as follows. *Abrus precatorius*: https://www.ncbi.nlm.nih.gov/bioproject/PRJNA510631; *Arabidopsis thaliana*: https://www.ncbi.nlm.nih.gov/bioproject/PRJNA116; *Carica papaya*: https://www.ncbi.nlm.nih.gov/bioproject/PRJNA264084; *Durio zibethinus*: https://www.ncbi.nlm.nih.gov/bioproject/PRJNA407962; *Glycine max*: https://www.ncbi.nlm.nih.gov/bioproject/PRJNA48389; *Herrania umbratica*: https://www.ncbi.nlm.nih.gov/bioproject/PRJNA389730; *Hevea brasiliensis*: https://www.ncbi.nlm.nih.gov/bioproject/PRJNA394253; *Manihot esculenta*: https://www.ncbi.nlm.nih.gov/bioproject/PRJNA394209; *Mucuna pruriens*: https://www.ncbi.nlm.nih.gov/bioproject/PRJNA414658; *Nicotiana attenuata*: https://www.ncbi.nlm.nih.gov/bioproject/PRJNA355166; *Oryza sativa*: https://www.ncbi.nlm.nih.gov/bioproject/PRJNA122; *Populus alba*: https://www.ncbi.nlm.nih.gov/bioproject/PRJNA638679; *Theobroma cacao*: https://www.ncbi.nlm.nih.gov/bioproject/PRJNA341501; *Vitis vinifera*: https://www.ncbi.nlm.nih.gov/bioproject/PRJNA399599; *Zea mays*: https://www.ncbi.nlm.nih.gov/bioproject/PRJNA358298. RNA-seq datasets of germinating seeds (at 0, 5, 10, 24 h), seedlings (leaf, stem and root), reproductive tissues (petal, stamen, pistil, calycle and flower) and developing seeds (at 0, 5, 10 and 20 days post anthesis) of *G. hirsutum* acc. TM-1 were downloaded from the NCBI SRA database under accession code PRJNA248163 (https://www.ncbi.nlm.nih.gov/bioproject/PRJNA248163/). RNA-seq in this study have been deposited at the National Center of Biotechnology Information under the accessions PRJNA706043 (https://www.ncbi.nlm.nih.gov/bioproject/PRJNA706043).
