# Supplementary material for: Suppression of GhGLU19 encoding β-1,3-glucanase promotes seed germination in cotton
Source: BMC Plant Biol. 2022 Jul 22;22:357. doi: 10.1186/s12870-022-03748-w (PMC9308338; doi:10.1186/s12870-022-03748-w)
Supplement: Supplementary file 1 — Additional file 1: Figure S1. Structure and characterization of GhGLU19. [file 12870_2022_3748_MOESM1_ESM.pdf]

A

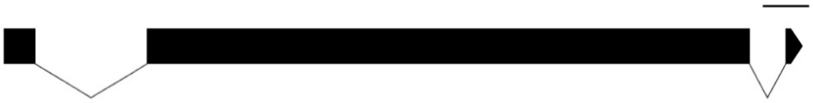

B

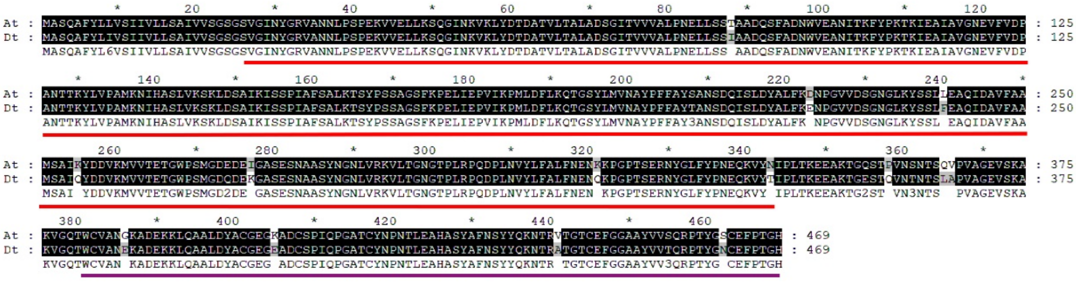

C

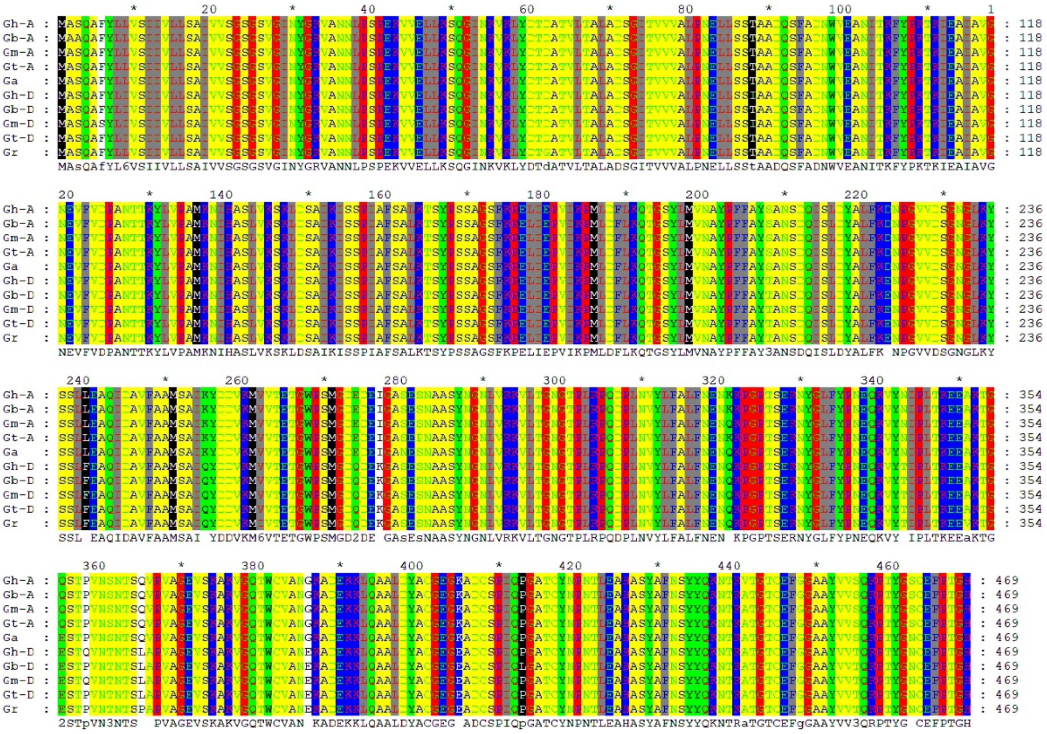

Figure S1 Structure and characterization of *GhGLU19*

(A) Genomic structure of *GhGLU19* (*Gh_A04G0109*). The gene length was 1,410 bp with three exons and two introns. Scale bar indicates 100 bp.

(B) Sequence alignment of GhGLU19 from A-subgenome and D-subgenome in *G. hirsutum* TM-1. The alignment was completed by ClustalX (v1.81). The homologous GhGLU19-A (Gh_A04G0109) and GhGLU19-D (Gh_D05G3612) were 98% identical with amino acid sequences. Conserved domain of Glycosyl hydrolase family 17 was underlined in red. The X8 domain involved in carbohydrate binding at the C terminus of family 17 glycosyl hydrolases, was indicated in purple.

(C) Sequence alignment of GhGLU19 homologs from different cotton species. The result showed that amino acid sequence of GLU19 was highly conserved in *G. hirsutum* (Gh_A04G0109 and Gh_D05G3612), *G. tomentosum* (Gotom.A04G015400 and Gotom.D05G439300), *G. barbadense* (Gobar.A04G013600 and Gobar.D05G415700), *G. mustelinum* (Gomus.A04G014100 and Gomus.D05G430600), *G. arboreum* (Ga05G4167) and *G. raimondii* (Gorai.009G442700). The sequences of different cotton species were downloaded from the CottonGen database (https://www.cottongen.org/data/download).
