# Supplementary material for: Suppression of GhGLU19 encoding β-1,3-glucanase promotes seed germination in cotton
Source: BMC Plant Biol. 2022 Jul 22;22:357. doi: 10.1186/s12870-022-03748-w (PMC9308338; doi:10.1186/s12870-022-03748-w)
Supplement: Supplementary file 2 — Additional file 2: Figure S2. Phylogenetic analysis of GLUs from G. hirsutum and other plants. [file 12870_2022_3748_MOESM2_ESM.pdf]

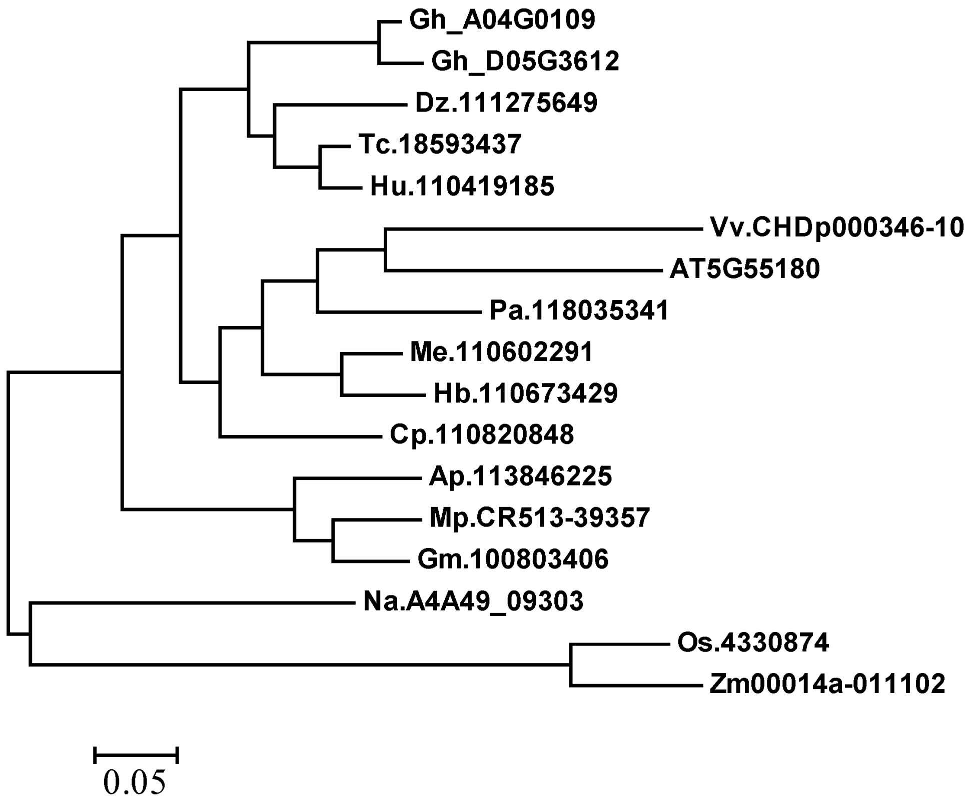

Figure S2 Phylogenetic analysis of GLUs from *G. hirsutum* and other plants

The phylogenetic tree was drawn by Maximum Likelihood method using the MEGA5.02. *G. hirsutum* (Gh), *Durio zibethinus* (Dz), *Theobroma cacao* (Tc), *Herrania umbratica* (Hu), *Carica papaya* (Cp), *Manihot esculenta* (Me), *Hevea brasiliensis* (Hb), *Populus alba* (Pa), *Vitis vinifera* (Vv), *Arabidopsis thaliana* (At), *Abrus precatorius* (Ap), *Mucuna pruriens* (Mp), *Glycine max* (Gm), *Artemisia annua* (Aa), *Helianthus annuus* (Ha), *Nicotiana attenuata* (Na), *Oryza sativa* (Os) and *Zea mays* (Zm). The sequences were downloaded from the National Center of Biotechnology Information (NCBI, http://www.ncbi.nlm.nih.gov/).
