# Supplementary material for: Suppression of GhGLU19 encoding β-1,3-glucanase promotes seed germination in cotton
Source: BMC Plant Biol. 2022 Jul 22;22:357. doi: 10.1186/s12870-022-03748-w (PMC9308338; doi:10.1186/s12870-022-03748-w)
Supplement: Supplementary file 3 — Additional file 3: Figure S3. Identification of GhGLU19-overexpressing and GhGLU19-suppressing transgenic cotton lines. [file 12870_2022_3748_MOESM3_ESM.pdf]

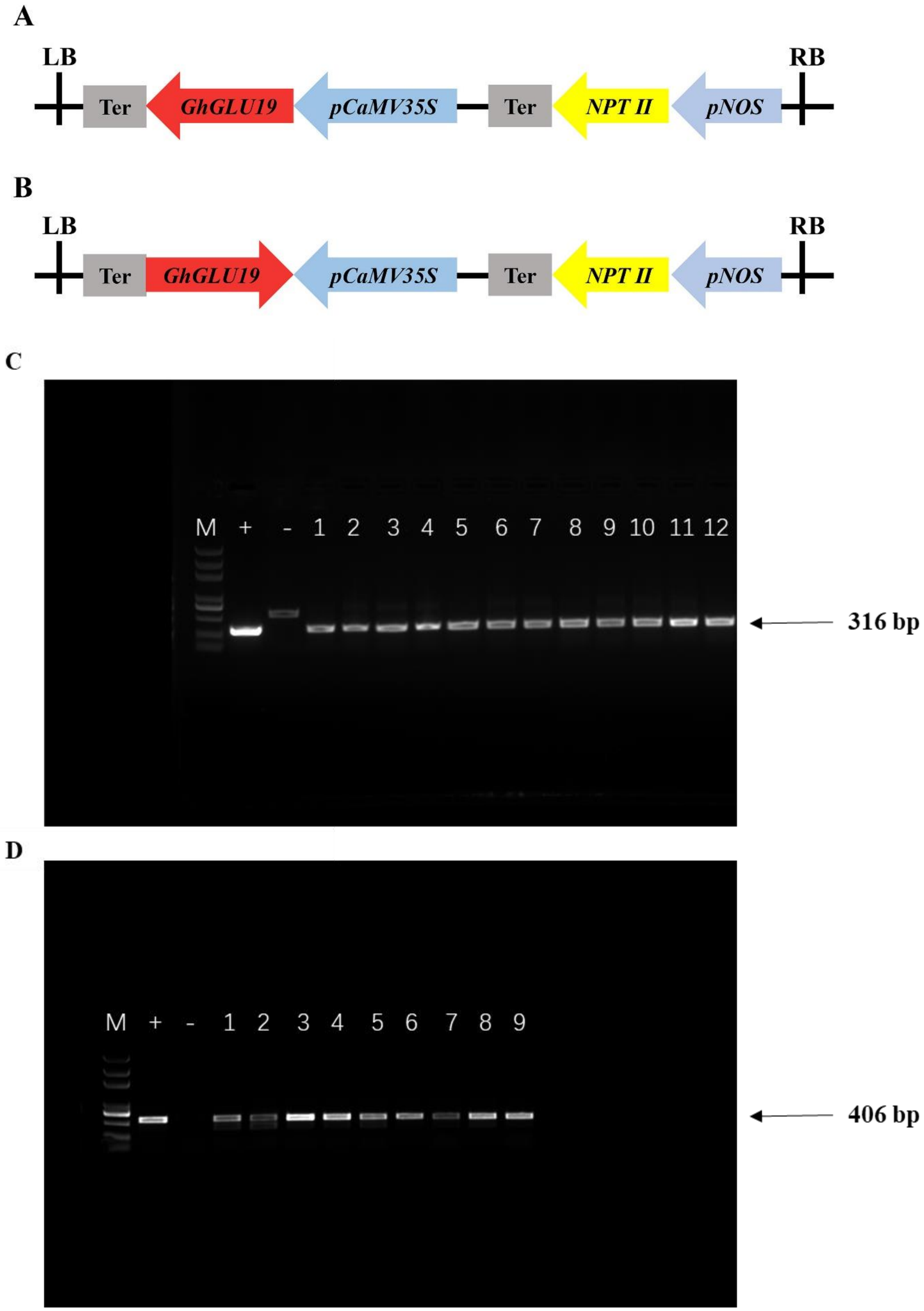
A
LB
Ter
GhGLU19
pCaMV35S
Ter
NPT II
pNOS
RB
B
LB
Ter
GhGLU19
pCaMV35S
Ter
NPT II
pNOS
RB
C
M + - 1 2 3 4 5 6 7 8 9 10 11 12
316 bp
D
M + - 1 2 3 4 5 6 7 8 9
406 bp

E

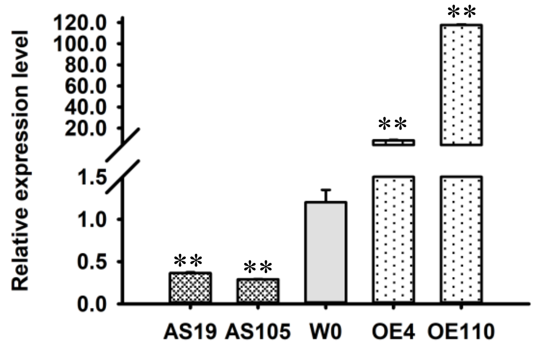

Figure S3. Identification of *GhGLU19*-overexpressing and *GhGLU19*-suppressing transgenic cotton lines

(A-B) Schematic plot of constructs for overexpression (A) and suppression (B) of *GhGLU19*. Full-length sequence and antisense sequence of *GhGLU19* ORF was cloned into *pBI121* vector and driven by constitutive promoter CaMV *35S*.

(C-D) Verification of transgenic events of overexpression (C) and suppression (D) of *GhGLU19* using PCR detection. + positive control, - negative control. Numbers present transgenic plants. (E) Relative expression level of *GhGLU19* in 2 h imbibed seeds of transgenic lines and control. AS19 and AS105 were *GhGLU19*- suppressing lines. OE4 and OE110 were *GhGLU19*-overexpressing lines. W0 was receptor. The data analysis was performed by Student's *t*-test (n = 3), ** p < 0.01.
