# Supplementary material for: Suppression of GhGLU19 encoding β-1,3-glucanase promotes seed germination in cotton
Source: BMC Plant Biol. 2022 Jul 22;22:357. doi: 10.1186/s12870-022-03748-w (PMC9308338; doi:10.1186/s12870-022-03748-w)
Supplement: Supplementary file 8 — Additional file 8: Figure S4. Expression heat map of genes encoding alcohol dehydrogenase and lactate dehydrogenase in transgenic and control imbibed seeds. [file 12870_2022_3748_MOESM8_ESM.pdf]

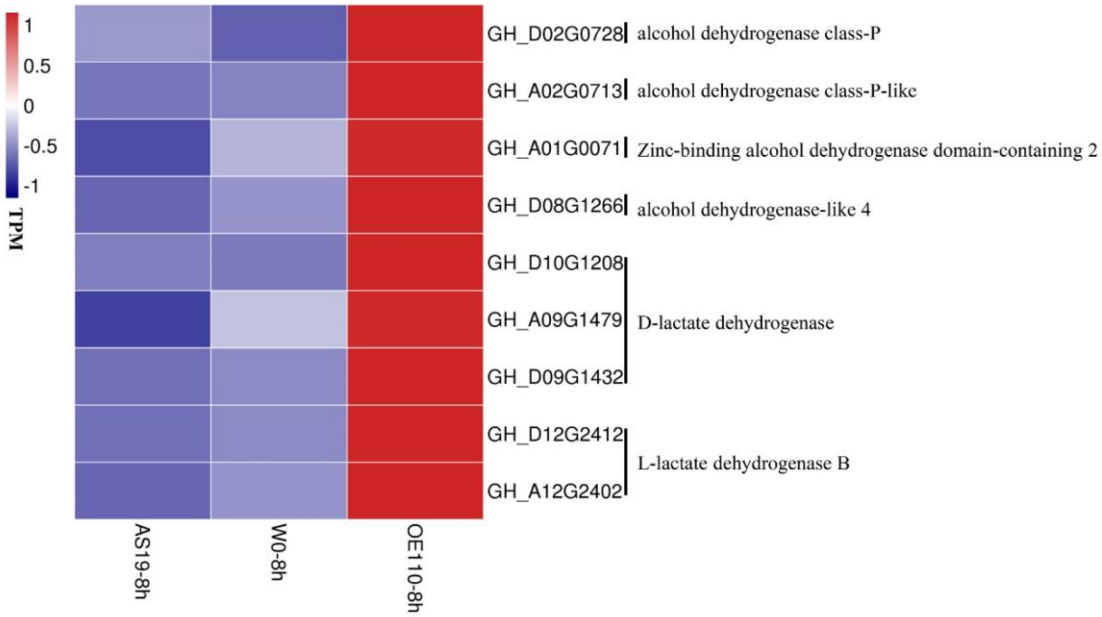

Figure S4 Expression heat map of genes encoding alcohol dehydrogenase and lactate dehydrogenase in transgenic and control imbibed seeds.
