# Supplementary material for: Suppression of GhGLU19 encoding β-1,3-glucanase promotes seed germination in cotton
Source: BMC Plant Biol. 2022 Jul 22;22:357. doi: 10.1186/s12870-022-03748-w (PMC9308338; doi:10.1186/s12870-022-03748-w)
Supplement: Supplementary file 10 — Additional file 10: Table S1. Primers used in this study. [file 12870_2022_3748_MOESM10_ESM.pdf]

Table S1 Primers used in this study

**Description**	**primer name**	**Sequence (5'-3')**	**Gene symbol**	**Accession number**
Over-expression vector construction	OE-GhGLU19-F	GCTCTAGAATGGCTTCCCAGGCGTTTTATC	*GhGLU19-A*	Gh_A04G0109
	OE-GhGLU19-R	CGAGCTCTCAATGCCCTGTTGGAAACTC		
Antisense vector construction	AS-GhGLU19-F	CGAGCTCATGGCTTCCCAGGCGTTTTATC	*GhGLU19-A*	Gh_A04G0109
	AS-GhGLU19-R	GCTCTAGATCAATGCCCTGTTGGAAACTC		
PCR amplification for transgenic plants	PCR-OE-F	CACAATCCCACTATCCTTCG		
	PCR-OE-R	ATTAGGAAGTGCGACGACCAC		
	PCR-AS-F	CACAATCCCACTATCCTTCG		
	PCR-AS-R	ACGAGTCAGGTTCCGGTAGCC		
Recombination vector construction	pET30-GhGLU19-F	GCCATGGCTGATATCGGATCCTCGGGTTCGGTCGGGATTAACT	*GhGLU19-A*	Gh_A04G0109
	pET30-GhGLU19-R	GTGGTGGTGGTGGTGCTCGAGATGCCCTGTTGGAAACTCACAACT		
qRT-PCR for GhGLU19	qRT-PCR-GhGLU19-F	AGAGGGTAAGGCTGATTGTAG	*GhGLU19*	Gh_A04G0109 Gh_D05G3612
	qRT-PCR-GhGLU19-R	CATATGTAGGACGTTGGGAGA		
qRT-PCR for *tubulin* as reference gene	qRT-PCR-tublin-F	CCGTAAACCTTATTCCATTCCC	*tublin*	KF555285.1
	qRT-PCR-tublin-R	AAGACCGCTGATGCTGTGAG		
qRT-PCR for *Histone3* as reference gene	qRT-PCR- *Histone3*-F	CGGTGGTGTGAAGAAGCCTCAT	*Histone3*	AF024716
	qRT-PCR- Histone3-R	AATTTCACGAACAAGCCTCTGGAA		
qRT-PCR for expression of genes in glycolysis and pyruvate pathway	GH_A06G0746-F	TTATATGATCCGTGCTAT	*PFK*	GH_A06G0746
	GH_A06G0746-R	TGTTATTACTACCTTGTTC		GH_D06G0723
	GH_A05G3421-F	TTATATTTGAGGGCATCTTACT	*ALDO*	GH_A05G3421
	GH_A05G3421-R	CAGCATTGTGAGCGTATA		GH_D05G3338
	GH_A09G1595-F	AGTGATGCTATTCTTCTT	*PK*	GH_A09G1595
	GH_A09G1595-R	AATATAGGTCTTGGTTGAA		GH_D09G1539
	GH_A12G1330-F	CACTATGCTGCTAGAGAT	*PDH*	GH_A12G1330
	GH_A12G1330-R	CTACCACCTCATCTATCTT		
qRT-PCR for expression of genes related to MVA and ABA biosynthesis	GH_D03G1746-F	AACCAACTACCTTGATAC	*HMGCS*	GH_D03G1746
	GH_D03G1746-R	CACTTCCGTAAGAGAATA		GH_A03G0218
	GH_A11G3372-F	TGCTGTGGATGATCTATA	*MVK*	GH_A11G3372
	GH_A11G3372-R	GCTTATACTTCATTGATGTC		GH_D11G3388

qRT-PCR for expression of genes involved in ABA signaling	GH_A12G2255-F	TTGTTAGTTAGGGAAGAC	*MVAK*	GH_A12G2255
	GH_A12G2255-R	TTGATGTTGACTGAAGAA		GH_D12G2272
	GH_A05G1770-F	TTGTTAGTTAGGGAAGAC	*FDPS*	GH_A05G1770
	GH_A05G1770-R	TTGATGTTGACTGAAGAA		GH_D05G1803
	qRT-PCR-ABA2-F	TAACGCAATTTTGACCCC	*ABA2*	GH_D01G2293
	qRT-PCR-ABA2-R	GAAACCGCAAGCATCTCC		GH_A01G2208
	GH_D10G0397-F	GTGCTAGAGTCGTATGTG	*PYL6*	GH_D10G0397
	GH_D10G0397-R	GCCAATCCTTCTGTTACC		GH_A10G0381
	GH_A11G0999-F	AGGCTCTTATCCGATGTAAT	*PYL9*	GH_A11G0999
	GH_A11G0999-R	ATCCGTTGATAGGCTCTG		
	GH_A09G1844-F	AAGTAGGTATCCAGGTTC	*PP2C*	GH_A09G1844
	GH_A09G1844-R	GGTTATGGTTATGGTCTTG		GH_D09G1798
	GH_D11G2017-F	GCAGAGTATTGATACGAT	*SnRK2*	GH_D11G2017
	GH_D11G2017-R	CAAGGTTGTCTACCATTA		GH_A11G1981
	qRT-PCR-ABI3-F	TGGAAGACATTGGAACAT	*ABI3*	GH_D07G1687
	qRT-PCR-ABI3-R	AGCAGCAGCAATTAGTAT		GH_A07G1687
	GH_D04G0072-F	TGATGACAAGGTTGAGACT	*ABI5*	GH_D04G0072
	GH_D04G0072-R	CAGCCACTAGGAGATGAAT		GH_A05G4302
